# Adjustments to Photosystem Stoichiometry and Electron Transfer Proteins Are Key to the Remarkably Fast Growth of the Cyanobacterium *Synechococcus elongatus* UTEX 2973

**DOI:** 10.1128/mBio.02327-17

**Published:** 2018-02-06

**Authors:** Justin Ungerer, Po-Cheng Lin, Hui-Yuan Chen, Himadri B. Pakrasi

**Affiliations:** a Department of Biology, Washington University, St. Louis, Missouri, USA; b Department of Energy, Environmental and Chemical Engineering, Washington University, St. Louis, Missouri, USA; Oregon State University

**Keywords:** cyanobacteria, electron transport, photosynthesis, *Synechococcus*

## Abstract

At the genome level, *Synechococcus elongatus* UTEX 2973 (*Synechococcus* 2973) is nearly identical to the model cyanobacterium *Synechococcus elongatus* PCC 7942 (*Synechococcus* 7942) with only 55 single nucleotide differences separating the two strains. Despite the high similarity between the two strains, *Synechococcus* 2973 grows three times faster, accumulates significantly more glycogen, is tolerant to extremely high light intensities, and displays higher photosynthetic rates. The high homology between the two strains provides a unique opportunity to examine the factors that lead to increased photosynthetic rates. We compared the photophysiology of the two strains and determined the differences in *Synechococcus* 2973 that lead to increased photosynthetic rates and the concomitant increase in biomass production. In this study, we identified inefficiencies in the electron transport chain of *Synechococcus* 7942 that have been alleviated in *Synechococcus* 2973. Photosystem II (PSII) capacity is the same in both strains. However, *Synechococcus* 2973 exhibits a 1.6-fold increase in PSI content, a 1.5-fold increase in cytochrome *b*_6_*f* content, and a 2.4-fold increase in plastocyanin content on a per cell basis. The increased content of electron carriers allows a higher flux of electrons through the photosynthetic electron transport chain, while the increased PSI content provides more oxidizing power to maintain upstream carriers ready to accept electrons. These changes serve to increase the photosynthetic efficiency of *Synechococcus* 2973, the fastest growing cyanobacterium known.

## IMPORTANCE

As the global population increases, the amount of arable land continues to decrease. To prevent a looming food crisis, crop productivity per acre must increase. A promising target for improving crop productivity is increasing the photosynthetic rates in crop plants. Cyanobacteria serve as models for higher plant photosynthetic systems and are an important test bed for improvements in photosynthetic productivity. In this study, we identified key factors that lead to improved photosynthetic efficiency and increased production of biomass of a cyanobacterium. We suggest that the findings presented herein will give direction to improvements that may be made in other photosynthetic organisms to improve photosynthetic efficiency.

## INTRODUCTION

The global population is increasing, while the demand for biofuels consumes more and more arable land that would otherwise be used for agriculture. These trends create a looming food crisis that will be realized within the next 50 years. In order to avoid such a crisis, global food production must increase even though the amount of farm land is decreasing. Over the past 50 years, production per hectare has more than doubled due to advances in controlling nutrition, pests, disease, and drought and increases in the amount of biomass partitioned into grain. Unfortunately, these problems are essentially solved which leaves little room for improvement in these areas in the future. Plants operate at ~1% of the theoretical maximum for solar energy capture and conversion which suggests that improvements to photosynthesis is a promising path for improving crop yields in the future.

Cyanobacteria are the ancient ancestors of the chloroplast and serve as a genetically tractable model for the study of photosynthesis. One model cyanobacterium, *Synechococcus elongatus* PCC 7942 (*Synechococcus* 7942), has a recently discovered relative, *Synechococcus elongatus* UTEX 2973 (*Synechococcus* 2973), that is nearly genetically identical but exhibits photosynthesis rates more than twofold higher. The two strains differ by only 55 single nucleotide polymorphisms (SNPs), a 7.5-kb deletion/insertion, and a 188-kb inversion; however, *Synechococcus* 2973 produces biomass at three times the rate of *Synechococcus* 7942 (see below). Unlike many model strains, *Synechococcus* 2973 is capable of biomass production at rates that are comparable to heterotrophs such as the yeast *Saccharomyces cerevisiae*. The extremely close relatedness of the two cyanobacterial strains offers an excellent system to examine how a slower-growing, less-productive model organism can be transitioned into a fast-growing and highly productive strain. We set out to compare the photophysiology of the two strains to elucidate which specific changes lead to the increased productivity of *Synechococcus* 2973. Such a comparison will give direction to the changes that must be made to improve the photosynthetic rates of other model and industrial relevant cyanobacteria and eventually higher plants.

## RESULTS

### Growth of *Synechococcus* 2973 and *Synechococcus* 7942.

**FIG 1:**
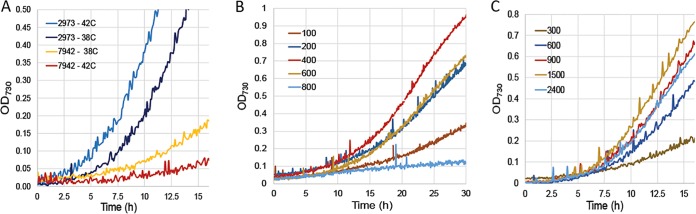
Growth of *Synechococcus* 7942 and *Synechococcus* 2973 under various conditions. (A) Comparison of growth at 38°C versus 42°C. *Synechococcus* 7942 was grown with 400 μmol m^−2^ s^−1^ light and 5% CO_2_, and *Synechococcus* 2973 was grown with 900 μmol m^−2^ s^−1^ light and 5% CO_2_. (B) Growth of *Synechococcus* 7942 at various light intensities at 38°C and 5% CO_2_. (C) Growth of *Synechococcus* 2973 at various light intensities at 38°C and 5% CO_2_. Light intensity in panels B and C is given in μmol m^−2^ s^−1^. Notice the difference in the scale of the *x* axis in panel B versus panels A and C.

Despite being genetically similar, the two *Synechococcus* strains display significant phenotypic differences such as optimum growth conditions and maximum growth rates under such conditions. In our previous study, we reported that *Synechococcus elongatus* UTEX 2973 grew best at 500 μmol m^−2^ s^−1^ light (20), which was the maximum intensity that our bioreactor could achieve. With an upgraded bioreactor (MC-1000HL; Photon Systems Instruments, Brno, Czech Republic), we returned to examine a wider range of growth conditions. We found that *Synechococcus elongatus* PCC 7942 grows best at 38°C and exhibits reduced growth at 42°C (Fig. 1A). In contrast, the optimum growth temperature for *Synechococcus* 2973 is 42°C, and it grows slightly slower at 38°C (Fig. 1A). Another characteristic difference between the two strains is their capacity for light tolerance and maximum growth rates under optimum conditions. *Synechococcus* 7942 achieves its maximum growth rate, a 4.9-h doubling time, at 400 μmol m^−2^ s^−1^ light, 5% CO_2_, and 38°C (Fig. 1B). Increasing light intensity above 400 μmol m^−2^ s^−1^ slows growth due to photoinhibition. In contrast, *Synechococcus* 2973 achieves the remarkable doubling time of 1.5 h at 1,500 μmol m^−2^ s^−1^ light, 5% CO_2_, and 42°C (Fig. 1C). Furthermore, *Synechococcus* 2973 does not appear to suffer from significant photoinhibition at light intensities that far exceed natural sunlight, up to 2,400 μmol m^−2^ s^−1^ (Fig. 1C).

Although they are closely related, *Synechococcus* 2973 and *Synechococcus* 7942 are two different strains. As such, they grow well under different conditions, and therefore, there is no single growth condition that both strains can be cultured under for direct comparison. Under the maximum light intensity that *Synechococcus* 7942 grows at (400 μmol m^−2^ s^−1^ light), both strains grow at similar rates, and we would not be able to investigate the rapid growth phenotype of *Synechococcus* 2973. If we grow both strains at higher light intensities, then *Synechococcus* 7942 becomes photoinhibited, and we would be comparing a strain experiencing severe photodamage to a healthy one. Therefore, to compare the two strains in this study, we chose conditions that work well for each strain. To limit the variables that are different in the growth conditions, we chose 38°C as the growth temperature because both strains grow well at this temperature. However, *Synechococcus* 7942 was cultured at 300 μmol m^−2^ s^−1^ light, while *Synechococcus* 2973 was cultured at 900 μmol m^−2^ s^−1^ light. The cyanobacteria grown under both light conditions were supplemented with 5% CO_2_.

### Glycogen accumulation.

**FIG 2:**
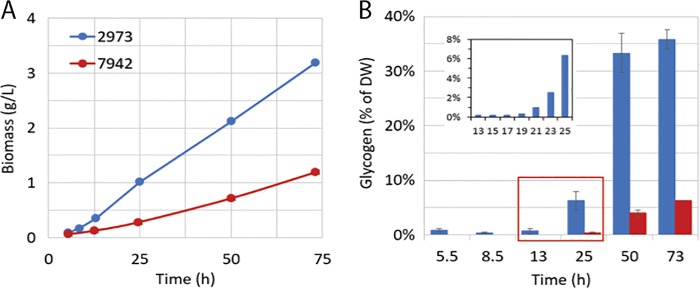
Biomass accumulation (A) and glycogen content (B) of *Synechococcus* 2973 and *Synechococcus* 7942. The inset graph in panel B presents glycogen synthesis between 13 and 25 h. DW, dry weight (cell weight).

*Synechococcus* 2973 demonstrates high growth rates under high light and high CO_2_ conditions. To understand more about carbon utilization by this fast-growing strain, we compared the time course of biomass and glycogen accumulation that occurs after log-phase growth has transitioned to linear growth at an optical density at 730 nm (OD_730_) of ~0.4. *Synechococcus* 2973 undergoes a protracted period of linear growth where it reached densities much higher than our bioreactor can record. During this time, *Synechococcus* 2973 accumulates biomass steadily at a rate of 1.1 g liter^−1^ day^−1^ (Fig. 2A), which is nearly three times higher than the rate shown by *Synechococcus* 7942, which accumulates biomass at a rate of 0.45 g liter^−1^ day^−1^. During the fast growth phase of *Synechococcus* 2973 (before 12 h), the glycogen content is extremely low (<1% of cell weight [dry weight {DW}]) (Fig. 2B). After that time, cells enter a linear growth phase (Fig. 2A). The glycogen content drastically increases between 19 and 25 h, changing 21-fold from 0.3% to 6.3% of DW (Fig. 2B, inset graph; also see Fig. S1 in the supplemental material). The amount of glycogen increases from 6% of DW (66 mg liter^−1^) to 33% of DW (693 mg liter^−1^) within the next 24-h span (24 h to 48 h) and ultimately reaches 1.1 g liter^−1^ (36% of DW) by day 3 (Fig. 2B). These results reveal that *Synechococcus* 2973 directs all of its fixed carbon into growth during log phase and then rapidly transitions into directing the high flux of carbon into storage during the linear growth phase. As for *Synechococcus* 7942, the biomass and glycogen contents accumulated at much lower rates. The glycogen content is negligible during the first day of growth, and it is less than 10% (75 mg liter^−1^) of DW at day 3 (Fig. 2B).

10.1128/mBio.02327-17.1 FIG S1  *Synechococcus* 2973 glycogen content at different growth phases. Download FIG S1, TIF file, 0.04 MB. Copyright © 2018 Ungerer et al. 2018 Ungerer et al. This content is distributed under the terms of the Creative Commons Attribution 4.0 International license.

### Pigment content.

**FIG 3:**
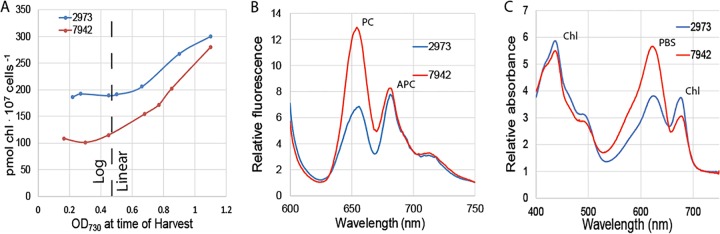
Pigment content of *Synechococcus* 2973 versus *Synechococcus* 7942. (A) Chlorophyll content as a function of culture density. As indicated, log growth occurs when the OD_730_ of the cell culture was below 0.4 and linear growth occurs when the OD_730_ of the cell culture was above 0.4. (B) 77 K fluorescence of *Synechococcus* 2973 versus *Synechococcus* 7942 with excitation at 590 nm. PC, phycocyanin; APC, allophycocyanin. (C) Absorption spectra of *Synechococcus* 2973 versus *Synechococcus* 7942. PBS, phycobilisome.

In this study, we performed several experiments that are typically normalized to chlorophyll content. When harvesting cells at similar densities, we observed large differences in chlorophyll content between samples for the two *Synechococcus* strains. The differences in chlorophyll content were also apparent in absorbance scans (Fig. 3C). If the strains differ in chlorophyll content, then normalization to chlorophyll will be an invalid method of comparison. We compared chlorophyll (Chl) content in the two strains and found that *Synechococcus* 2973 has 190 ± 7 pmol Chl 10^7^ cells^−1^ s^−1^ compared to 112 ± 9 pmol Chl 10^7^ cells^−1^ s^−1^ in *Synechococcus* 7942 during log-phase growth (Fig. 3A) (1). Our results show that during log-phase growth, *Synechococcus* 2973 has a 1.7-fold-higher chlorophyll content per cell compared to *Synechococcus* 7942; however, the chlorophyll content becomes more similar as the cultures enter linear growth, differing by only 1.1-fold near stationary phase. We also noted that in both strains, chlorophyll content per cell remains constant during log growth but increases as a function of culture density during linear growth. This highlights the importance of harvesting cultures during log phase when normalizing to chlorophyll content, as chlorophyll is variable during linear growth. Over the course of this study, normalization was calculated based on cell number to avoid errors caused by the different chlorophyll levels between the two strains.

*Synechococcus* 2973 is significantly less blue than *Synechococcus* 7942 (Fig. S2), indicating that *Synechococcus* 2973 exhibits an altered pigment composition. To examine the pigment compositions of the two strains, we determined room temperature absorption spectra from 400 nm to 750 nm. The absorbance scans were taken of cultures with equal cell numbers and normalized at 750 nm. We observed increased absorbance at 680 nm in *Synechococcus* 2973 that resulted from its increased chlorophyll content (Fig. 3C). We also observed significantly reduced phycobilisome absorbance at 625 nm, indicating that *Synechococcus* 2973 exhibits decreased phycobilisome content (Fig. 3C). We examined the difference using low-temperature fluorescence at 77 K with excitation at 590 nm to excite phycobilins and normalized at 750 nm. Phycocyanin (PC) exhibits a fluorescence maximum at 665 nm, and allophycocyanin (APC) exhibits a fluorescence maximum at 682 nm (2). We found that *Synechococcus* 2973 exhibits a significantly reduced phycocyanin fluorescence relative to *Synechococcus* 7942, while allophycocyanin fluorescence remains unchanged (Fig. 3B). In *Synechococcus* 2973, the ratio of relative fluorescence of PC/APC was 0.88, while it was 1.55 in *Synechococcus* 7942. Interestingly, the decrease in phycobilisome absorbance is a result of fewer or shorter rods (PC) rather than a reduction in the number of phycobilisome complexes, as indicated by the same fluorescence from the APC cores in both strains.

10.1128/mBio.02327-17.2 FIG S2  Color difference between *Synechococcus* 2973 and *Synechococcus* 7942. Download FIG S2, TIF file, 0.2 MB. Copyright © 2018 Ungerer et al. 2018 Ungerer et al. This content is distributed under the terms of the Creative Commons Attribution 4.0 International license.

### Photosynthetic parameters.

**FIG 4:**
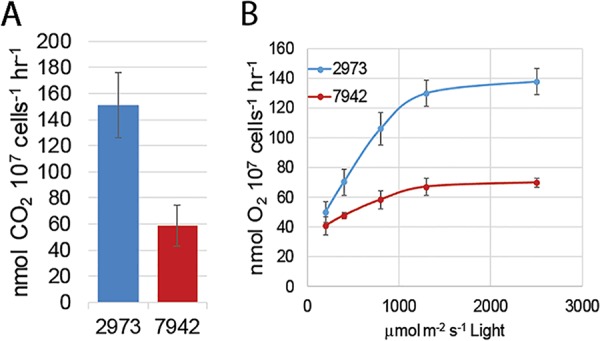
Photosynthetic parameters of *Synechococcus* 2973 and *Synechococcus* 7942. (A) CO_2_ uptake rates. (B) Whole-chain O_2_ evolution at various light intensities.

We set about characterizing the photosynthetic parameters that contribute to rapid autotrophic growth. To generate biomass at a higher rate than *Synechococcus* 7942, *Synechococcus* 2973 must acquire more fixed carbon. We compared CO_2_ uptake rates for the two strains and found that *Synechococcus* 2973 takes up carbon at a rate of 151 nmol CO_2_ 10^7^ cells^−1^ h^−1^ versus 59 nmol CO_2_ 10^7^ cells^−1^ h^−1^ for *Synechococcus* 7942 (Fig. 4A). It follows then that *Synechococcus* 2973 is also likely to exhibit higher photosynthetic rates to support higher carbon fixation rates. We compared whole-chain O_2_ evolution rates for the two strains using a Clarke electrode and found that *Synechococcus* 2973 also exhibits a twofold-higher maximum rate of O_2_ evolution, 139 ± 24 nmol O_2_ 10^7^ cells^−1^ h^−1^ versus 74 ± 9 nmol O_2_ 10^7^ cells^−1^ h^−1^ for *Synechococcus* 7942 (Fig. 4B). Under low light, the two strains differed by only 24% which mirrors the similar growth rates that are observed at low light intensities. As light intensity increases, the photosynthetic electron transport rate of *Synechococcus* 2973 increases much more rapidly than that of *Synechococcus* 7942, culminating in a twofold difference in the O_2_ evolution rate. We also examined respiratory O_2_ uptake in both strains. It was found that *Synechococcus* 2973 exhibits a 2.4-fold-higher rate of respiratory O_2_ uptake (14.3 nmol O_2_ 10^7^ cells^−1^ h^−1^ versus 5.9 nmol O_2_ 10^7^ cells^−1^ h^−1^). However, respiratory O_2_ uptake is only about 10% of photosynthetic O_2_ evolution and does not contribute significantly to total oxygen evolution rates.

### PSII activity.

**FIG 5:**
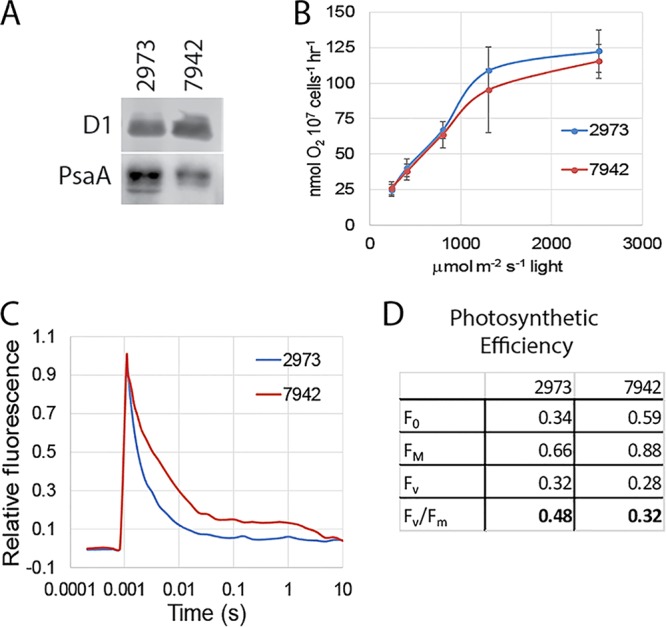
Function of PSII. (A) Western blot loaded based on equal cell number and probed with antibodies against PSII (D1) or PSI (PsaA). (B) PSII-mediated O_2_ evolution with DCMU and DBMIB. (C) Q_A_^−^ reoxidation kinetics of *Synechococcus* 2973 versus *Synechococcus* 7942. (D) Photosynthetic efficiencies of *Synechococcus* 2973 versus *Synechococcus* 7942.

We hypothesized that *Synechococcus* 2973 would have increased photosystem II (PSII) content to support the higher rate of photosynthetic O_2_ evolution that we recorded (Fig. 4B). Western blot analysis was used to compare the PSII content in the two strains. Equal cell numbers of each strain were harvested, and the chlorophyll content, which varied in the two strains, was measured. After the membranes were purified, we adjusted the samples to the same chlorophyll ratio that was measured in whole cells to maintain equal cell numbers for comparison. The Western blot indicates that both strains contain similar amounts of PSII on a per cell basis (Fig. 5A). Since both strains have similar PSII contents, we hypothesized that the PSII centers in *Synechococcus* 2973 must be more active than those in *Synechococcus* 7942. We next interrogated PSII activity directly by measuring PSII-mediated O_2_ evolution in the presence of saturating FeCN and 2,6-dichloro-*p*-benzoquinone (DCBQ) which accept electrons directly from PSII (2). Interestingly, we found that both strains had similar maximum capacities for PSII, as indicated by the similar rates of PSII-mediated O_2_ evolution (Fig. 5B).

Comparing whole-chain to PSII-mediated O_2_ evolution rates of the two strains shows that *Synechococcus* 2973 operates photosynthesis at close to its maximum capacity, while *Synechococcus* 7942 shows a significant inhibition of O_2_ evolution under real-world conditions. This suggests that *Synechococcus* 7942 has a photosynthetic bottleneck in the electron transport chain (ETC) downstream of PSII that is alleviated in *Synechococcus* 2973. If electron transport out from PSII is decreased due to a lack of carriers or acceptors, there will be a bottleneck because the cells cannot transfer electrons through the ETC fast enough to keep up with PSII. If no oxidized carrier is available to accept electrons from PSII, the reaction center will remain in the closed state for longer periods of time, and oxygen evolution will be decreased. While in the closed state, PSII reaction centers will release excess energy through chlorophyll fluorescence, and as PSII is reoxidized by plastoquinone, the reaction centers will reopen, and fluorescence will decrease (3). We used an FL-200 dual modulation PAM fluorometer (Photon Systems Instruments, Brno, Czech Republic) to compare primary quinone electron acceptor of reaction center II (Q_A_^−^) reoxidation kinetics of the two strains. After double normalization at minimum fluorescence (*F*_0_) and maximum fluorescence (*F*_*m*_), we found that the rate of Q_A_^−^ reoxidation is significantly higher in *Synechococcus* 2973 (halftime of 1.6 ms) compared to *Synechococcus* 7942 (halftime of 3.1 ms) (Fig. 5C). This suggests that *Synechococcus* 2973 has a higher whole-chain O_2_ evolution rate because it is better able to move electrons out of PSII as indicated by the increased rate of Q_A_^−^ reoxidation.

Using the unnormalized fluorescence data, we found that variable fluorescence (*F*_*v*_) was similar in both strains, which further supports both strains having similar PSII content. We also calculated the photosynthetic efficiency (*F*_*v*_/*F*_*m*_) of PSII for each strain (4). The *F*_*v*_/*F*_*m*_ value obtained from *Synechococcus* 2973 is 0.48 ± 0.03, while the value obtained from *Synechococcus* 7942 is 0.32 ± 0.05 (Fig. 5D). Both strains had similar maximum capacities for PSII; however, *Synechococcus* 2973 operates PSII more efficiently under real-world conditions as indicated by whole-chain O_2_ evolution and photosynthetic efficiency values. We suggest here that a downstream bottleneck in the ETC in *Synechococcus* 7942 causes a backup of electron flow which slows turnover of PSII, because reaction centers are stuck in the closed state for longer periods of time.

In *Synechococcus* 7942, the PSI/PSII ratio varies from 2 to 3.5 (5–7), and there are 96 chlorophyll molecules per PSI center compared to 35 chlorophyll molecules per PSII center; thus, most of the chlorophyll is found associated with PSI (8–10). Therefore, we hypothesized that the increased chlorophyll content would be associated with an increase in PSI content in *Synechococcus* 2973. We measured photoactive PSI content to estimate total PSI content per cell. A JTS-10 pump probe spectrophotometer (BioLogic Science Instruments) was used to measure maximum *A*_705_ of fully oxidized PSI after a saturating light pulse in the presence of dibromothymoquinone (DBMIB) and 3-(3′,4′-dichlorophenyl)-1,1-dimethylurea (DCMU) to block cyclic and linear electron flow. A molar extinction coefficient for P700^+^ of 70 mM^−1^ cm^−1^ was used to obtain the concentration of photo-oxidizable PSI in the sample (10). *Synechococcus* 2973 was found to have 1.95 ± 0.2 pmol PSI per 10^7^ cells, while *Synechococcus* 7942 has 1.19 ± 0.2 pmol PSI per 10^7^ cells (Table 1). Therefore, *Synechococcus* 2973 has 1.6-fold more PSI per cell, which suggests that the 1.7-fold increase in chlorophyll in *Synechococcus* 2973 results exclusively from increased PSI content. We also compared the PSI contents of the two strains by Western blotting and quantitated 1.7-fold-more PSI in *Synechococcus* 2973 than in *Synechococcus* 7942 by this method (Fig. 5A). Not all PSI centers may be photo-oxidizable, thus we validated our estimate by calculating the number of chlorophyll molecules per PSI center based on our measurement of the total amount of chlorophyll and number of PSI centers obtained. We calculated that *Synechococcus* 2973 had 97 chlorophyll molecules per PSI center, while *Synechococcus* 7942 had 100 chlorophyll molecules per PSI center (Table 1). There should be 96 chlorophyll molecules per PSI center plus a small amount of chlorophyll in PSII. Taking the PSI/PSII ratio into consideration, our estimation of PSI content is within 10% of the expected value based on chlorophyll content.

**TABLE 1:** PSI content of *Synechococcus* 2973 and *Synechococcus* 7942

*Synechococcus* strain	PSI content (pmol/10^7^ cells)	Chlorophyll content (pmol/10^7^ cells)	No. of chlorophyll molecules/PSI
2973	1.95	189	96.9
7942	1.19	119	100

### Cytochrome *f* and plastocyanin kinetics.

We have demonstrated that *Synechococcus* 2973 displays a higher photosynthetic rate than *Synechococcus* 7942 and that the increased rate is due to an ETC bottleneck downstream of PSII being alleviated. It is likely that increased PSI content leads to more efficient flow of electrons through the ETC by oxidizing upstream ETC carriers so that they may accept more electrons. To examine this in more detail, we studied cytochrome *f* and plastocyanin redox kinetics using a highly sensitive JTS-10 pump probe spectrophotometer.

**FIG 6:**
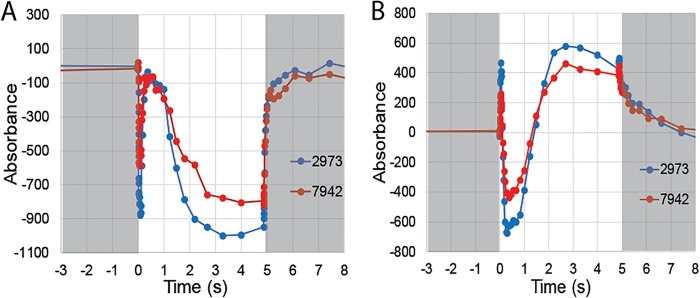
Cytochrome *b*_6_*f* and plastocyanin oxidation-reduction kinetics in *Synechococcus* 2973 and *Synechococcus* 7942. (A) Cytochrome *f* kinetics. (B) Plastocyanin kinetics. Dark (gray background) and light (white background) conditions are indicated in the figure.

For cytochrome *f*, absorption decreases as a function of oxidation. An initial drop in absorption upon illumination is due to P700^+^ pulling electrons off cytochrome *f* (through plastocyanin), followed by a rise in absorption at ~150 ms after illumination that is caused by the arrival of electrons from PSII. Absorption then falls again, as PSI draws off electrons faster than PSII can replenish them until the light is turned off, at which time cytochrome *f* returns to the dark-adapted state. We subjected samples that were adjusted to equal cell numbers to determine the oxidation-reduction kinetics of the cytochrome complex in the two cyanobacterial strains (Fig. 6A). Due to the increased PSI content of *Synechococcus* 2973, we observed a stronger initial oxidation of the cytochrome *f* pool upon illumination in this strain. Both strains have similar PSII contents, and both return the cytochrome *f* pool to the resting state with the initial pulse of electrons; however, the cytochrome pool became more oxidized once again after ~2-s illumination in *Synechococcus* 2973, because the increased concentration of P700^+^ has stronger oxidizing power for the plastocyanin pool, which in turn oxidizes the cytochrome *f* pool. Careful examination of these data reveals a bottleneck in the ETC. Immediately upon illumination, the oxidizing power of PSI generates a more oxidized cytochrome *f* pool in *Synechococcus* 2973. This creates a larger hole for the electrons to flow into and allows more influx of electrons from PSII. The initial pulse of electrons is sufficient to fully rereduce the cytochrome *f* pool in both strains; however, in *Synechococcus* 2973, the oxidized cytochrome *f* pool must accept more electrons to reach the fully reduced state. Since both strains have equal capacities for PSII, but the cytochrome *f* pool in *Synechococcus* 2973 can accept more electrons, higher electron flux through cytochrome *f* would be allowed in *Synechococcus* 2973.

From the same series of experiments, we also determined the kinetics of plastocyanin oxidation and reduction (Fig. 6B) (11, 12). In the case of plastocyanin, absorption increases with oxidation and decreases with reduction. An initial rise in absorption upon illumination is due to P700^+^ oxidizing the plastocyanin pool. As the initial burst of electrons arrives from PSII through cytochrome *f*, the absorption drops to below the initial level. Finally, the absorption increases again, as P700^+^ removes electrons from the plastocyanin pool faster than they are replenished. Comparing cytochrome *f* and plastocyanin kinetics of the two strains at 300 ms after illumination reveals more about the bottleneck in the ETC. Plastocyanin is more deeply oxidized immediately upon illumination; it is more deeply reduced by electrons arriving from PSII 300 ms after illumination. At 300 to 800 ms, the cytochrome *f* pool is fully rereduced and cannot accept additional electrons in either strain, but the plastocyanin pool is more deeply reduced by the initial burst of electrons from PSII; thus, more electrons are passing through cytochrome *f* into plastocyanin in *Synechococcus* 2973 under steady-state conditions. Therefore, we conclude that the bottleneck in the ETC occurs as the electrons are passing through cytochrome *f*.

We repeated the experiment on cytochrome *f* and plastocyanin kinetics in the presence of the inhibitors DCMU, DBMIB, and methyl viologen (Fig. S3). This set of inhibitors prevents electrons from flowing into cytochrome *f* and plastocyanin while allowing both pools to become fully oxidized by PSI. After 2.5 s of saturating light, we collected the maximum absorbance and applied extinction coefficients of 18 mM cm^−1^ and 4.7 mM cm^−1^ for cytochrome *f* and plastocyanin, respectively, to determine the concentration of each within the cell (Fig. S2) (11, 13). We found that *Synechococcus* 2973 has 1.38 ± 0.1 pmol cytochrome *f* per 10^7^ cells, while *Synechococcus* 7942 has only 0.86 ± 0.1 pmol cytochrome *f* per 10^7^ cells. The plastocyanin contents were 2.21 ± 0.3 pmol per 10^7^ cells and 1.07 ± 0.2 pmol per 10^7^ cells for *Synechococcus* 2973 and *Synechococcus* 7942, respectively. Therefore, on a cellular basis, *Synechococcus* 2973 shows a 1.5-fold increase in cytochrome *f* and a 2.2-fold increase in plastocyanin. Increased contents of carriers in the electron transport chain allows higher flux of electrons through the ETC to complement the increased PSI capacity, which leads to higher photosynthetic rates in *Synechococcus* 2973.

10.1128/mBio.02327-17.3 FIG S3  Absorbance changes for cytochrome *f* (A) and plastocyanin (B) with DCMU, DBMIB, and methyl viologen (MV). Download FIG S3, TIF file, 0.1 MB. Copyright © 2018 Ungerer et al. 2018 Ungerer et al. This content is distributed under the terms of the Creative Commons Attribution 4.0 International license.

## DISCUSSION

*Synechococcus* 2973 and *Synechococcus* 7942 are nearly genetically identical strains. However, *Synechococcus* 2973 exhibits a 3-fold-higher growth rate and a 2.5-fold-higher rate of glycogen accumulation, which are the result of a 2.5-fold increase in carbon uptake and 1.9-fold-higher rate of O_2_ evolution, indicating higher photosynthetic rates in *Synechococcus* 2973. Both strains have similar PSII contents, and as such, the maximum photosynthetic capacity of PSII is the same in both strains. The higher photosynthetic rate is the result of a 1.5-fold increase in photosynthetic efficiency in *Synechococcus* 2973. The increase is attributable to a 1.9-fold increase in the rate that electrons exit PSII (Q_A_^−^ reoxidation), a 1.6-fold increase in PSI content, a 1.5-fold increase in cytochrome *f* content, and a 2.2-fold increase in plastocyanin content. It is worth noting that all the aforementioned fold increases in *Synechococcus* 2973 correlate well with each other.

Both strains share the same capacity of PSII; however, *Synechococcus* 2973 displays a higher flux of electrons from water to CO_2_ as indicated by its higher carbon fixation rate and higher rate of whole-chain oxygen evolution. *Synechococcus* 7942 displays a marked bottleneck in the ETC that reduces flux from CO_2_ to water, reduces photosynthetic efficiency, and decreases photosynthetic rates under real-world conditions. This bottleneck is alleviated in *Synechococcus* 2973. The specific location of the bottleneck occurs as the electrons pass through the cytochrome *b*_6_*f* complex. Since both strains have the same capacity of PSII as indicated by PSII-mediated O_2_ evolution, both strains should be capable of sending a similar number of electrons from PSII under saturating light. However, this is not the case under real-world conditions. *Synechococcus* 2973 shows a higher flux of electrons out of PSII as indicated by the faster Q_A_^−^ reoxidation kinetics. Although the potential electron flux from PSII is similar for both strains, *Synechococcus* 2973 has a larger pool of downstream ETC carriers waiting to receive the electrons, which allows higher photosynthetic electron flux in the strain. An initial pulse of electrons fully returns cytochrome *f* back to the reduced state in both *Synechococcus* strains; however, *Synechococcus* 2973 accepts more reducing equivalents to reach the resting state, because the oxidized cytochrome *f* pool is larger. Since the same number of electrons could leave PSII in both strains, but more electrons can be accepted by cytochrome *f* in *Synechococcus* 2973, we conclude that the unaccepted electrons back up in the ETC in *Synechococcus* 7942. The lack of available oxidized carriers in the ETC limits the electrons from moving on from PSII. The reduced flux of electrons out of PSII was duly noted in our measurements of Q_A_^−^ reoxidation kinetics. The backed-up electrons cause the PSII reaction centers to remain in the closed state for longer periods of time, which reduces the photosynthetic efficiency and results in a lower rate of O_2_ evolution under real-world conditions. It is likely that this also increases the propensity of photodamage in *Synechococcus* 7942, making it sensitive to high light intensities.

*Synechococcus* 2973 overcomes the bottleneck by displaying both increased levels of electron carriers in the ETC and increased PSI content, which serves to pull more electrons through the ETC. Although we were not able to address plastoquinone levels, we conclude that they are sufficiently high to not impede electron flow, because we observed that the cytochrome *f* pool becomes fully rereduced to the dark-adapted state by the initial pulse of electrons from PSII in both strains. In *Synechococcus* 2973, the increased levels of cytochrome *f* and plastocyanin serve to allow a larger volume of electron flux through the ETC. The increased level of PSI serves to oxidize the increased levels of electron carriers so that they can accept electrons. Together, these two features result in increased light-driven electron transfer rates which drive a higher rate of carbon fixation, thus increasing photosynthetic efficiency in *Synechococcus* 2973. The ability of *Synechococcus* 2973 to empty electrons out of PSII more efficiently and rapidly would also serve to reduce photodamage by reducing the propensity for charge recombination that exists while reaction centers are stuck in the closed state. Therefore, the more efficient electron transport chain would increase light tolerance by reducing the rate of photodamage in *Synechococcus* 2973. It is interesting that *Synechococcus* 7942 has the PSII capacity to support a much higher rate of photosynthesis, but the lack of downstream carriers creates a bottleneck in the ETC that suppresses the photosynthetic rate. It has also been previously shown that overexpression of plastocyanin from *Anabaena* in *Synechococcus* 7942 increases electron transport ~2.5-fold (14). Cyanobacteria are recognized for their high PSI/PSII ratio in conjunction with high photosynthetic efficiencies compared to higher plants. In this study, we found that further increasing this ratio by 1.7-fold generated a significant boost in photosynthetic efficiency as well as productivity. *Synechococcus* 2973 operates PSII at near maximum capacity, while *Synechococcus* 7942 and other widely used cyanobacteria such as *Synechocystis* sp. strain PCC 6803 operate PSII at well below their maximum capacity. It is also worth noting that *Synechococcus* 2973 seems specifically adapted to a high light environment. It shows a significant reduction in phycobilisome antennae, which are less important under saturating light but useful under low light. Phycobilisomes are costly to make, and their reduction may free up resources for the increased production of other photosynthetic components such as more PSI and higher levels of ETC carriers. Interestingly, the observations reported here are predicted by the autotrophic replicator model (ARM) where the autotrophic growth rate is formulated as a proteome allocation optimization problem. This model predicts that increased growth rate would be associated with increased allocation of proteins to photosynthetic electron transport at the expense of protein resources being allocated to light-harvesting complexes (15).

The results presented in this study suggest a strategy for improving photosynthetic efficiency and ultimately productivity in these and other cyanobacteria. It is commonly accepted that RuBisCO activity is the limiting factor in photosynthesis. Here we show that electron flux through the photosynthetic electron transport chain, and hence energy production, limits the photosynthetic rate in *Synechococcus* 7942. Future work will be needed to identify which of the 55 single nucleotide differences between the two organisms lead to the marked changes in the photosynthetic apparatus that we report here.

## MATERIALS AND METHODS

### Strains and growth conditions.

*Synechococcus elongatus* UTEX 2973 (*Synechococcus* 2973) and *Synechococcus elongatus* PCC 7942 (*Synechococcus* 7942) were maintained on BG11 agar plates at 38°C with 125 μmol m^−2^ s^−1^ light. *Synechococcus* 2973 was routinely grown in BG11 liquid medium at 38°C with 900 μmol m^−2^ s^−1^ light and 5% CO_2_ in an MC-1000 multicultivator (Photon Systems Instruments, Czech Republic). *Synechococcus* 7942 was routinely grown in BG11 liquid medium at 38°C with 400 μmol m^−2^ s^−1^ light and 5% CO_2_ in an MC-1000 multicultivator. For growth curves, strains were grown under the conditions indicated. The multicultivator was used to simultaneously grow eight cultures under different conditions and record the optical density at 730 nm (OD_730_) of the cultures every 5 min over the course of the growth experiment. Growth rates (*K*′) were calculated using Microsoft Excel by fitting an exponential curve to the logarithmic section of the growth data (typically OD_720_ of <0.3) and using the slope, *m*, as *K*′ (*y* = *ke^mx^*). Doubling times were then calculated as ln(2)/*K*′.

### Glycogen determination.

Cultures were collected by centrifugation, and the pellets were resuspended with 300 μl KOH (30% [wt/vol]) each and incubated at 95°C for 90 min. Glycogen was precipitated by adding 1.2 ml absolute ethanol, and the samples were kept on ice for 2 h. Glycogen was collected by centrifugation at full speed (16K × *g*) for 5 min. The pellets were washed twice with 1 ml absolute ethanol. The washed pellets were dried at 60°C for 15 min until the remaining ethanol was evaporated. The dried samples were resuspended in 300 μl sodium acetate solution (100 mM, pH 4.75). Then, glycogen was digested to glucose by amyloglucosidase (4 U/assay) for 25 min at 55°C. After digestion, the insoluble pellets were removed by centrifugation, and the supernatants were used for determining the glycogen content using a glucose (HK) assay kit (Sigma, USA). Samples that were not treated with amyloglucosidase were included to determine the background glucose content. By subtraction of the background glucose content, the actual glycogen concentration was determined.

### Thylakoid membrane preparation.

The *Synechococcus* 2973 and *Synechococcus* 7942 preparations with same cell numbers were harvested and broken by bead-beating technique (16). Thylakoid membranes were resuspended in RB (50 mM morpholineethanesulfonic acid [MES]–NaOH [pH 6.0], 10 mM MgCl_2_, 5 mM CaCl_2_, 25% glycerol) and mixed with β-d-dodecyl maltoside (DDM) to a final concentration of 1% DDM, and then incubated on ice in the dark for 30 min. Solubilized membranes were isolated by ultracentrifugation at gradually increasing speed from 120 to 27,000 × *g* at 4°C for about 20 min. The solubilized membranes were then stored at −80°C for further use.

### SDS-PAGE and immunoblot analysis.

The solubilized membranes of *Synechococcus* 2973 and *Synechococcus* 7942 were loaded on an equal cell number basis. Proteins were analyzed by SDS-PAGE as described previously (17). For the immunoblot analysis, the SDS-polyacrylamide gels were blotted onto polyvinylidene difluoride (PVDF) membranes. The membranes were then incubated with PsaA- and D1-specific primary antibodies, respectively, and then incubated with secondary antibodies (horseradish peroxidase [HRP]). The target proteins were detected and visualized by chemiluminescence on an ImageQuant LAS-4000 imager.

### Cell counting.

Cultures were grown in MC-1000 multicultivators at 38°C, 5% CO_2_, and with 900 or 400 μmol m^−2^ s^−1^ light for *Synechococcus* 2973 and *Synechococcus* 7942, respectively. Twenty microliters of culture was removed at various time points during growth, and cells were counted with an automated cell counter (Cellometer Vision; Nexcelom). The counted images were manually curated to improve accuracy of the counts. The accompanying Cellometer software reported cell counts in cells per milliliter after curation. The counts were used to construct an accurate six-point standard curve of each strain for cell number versus OD_730_ reported by the multicultivator for cultures ranging in densities from OD_730_ values of 0.1 to 1.0. The relationship was found to be linear over this entire range. The standard curve was used to convert OD_730_ to cell number in later experiments.

### Chlorophyll content.

Cultures were grown in MC-1000 multicultivators at 38°C, 5% CO_2_, and either 900 or 400 μmol m^−2^ s^−1^ light for *Synechococcus* 2973 and *Synechococcus* 7942, respectively. One milliliter of culture was removed at various time points during growth, and the chlorophyll content was determined by a methanol extraction method (1).

### 77 K fluorescence.

Cultures were grown in MC-1000 multicultivators at 38°C, 5% CO_2_, and either 900 or 400 μmol m^−2^ s^−1^ light for *Synechococcus* 2973 and *Synechococcus* 7942, respectively. The fluorescence emission spectra of phycobilisomes from whole cells of each strain were measured at 77 K with samples adjusted to equal cell number (2 × 10^8^ cells per ml). Excitation occurred at 590 nm, and fluorescence emission was recorded between 600 nm and 750 nm and normalized at 750 nm. The measurements were made on a SPEX fluoromax 2 spectrofluorimeter and analyzed with Data Max for Windows.

### Absorption spectra.

Cultures were grown in MC-1000 multicultivators at 38°C, 5% CO_2_, and either 900 or 400 μmol m^−2^ s^−1^ light for *Synechococcus* 2973 and *Synechococcus* 7942, respectively. Absorption spectra of *Synechococcus* 2973 and *Synechococcus* 7942 that were harvested during log phase were determined on an Olis DW-2000 spectrophotometer, and data were analyzed with Olis Globalworks software. Spectra were normalized at 750 nm to correct for differences in light scattering.

### CO_2_ uptake rate.

*Synechococcus* 2973 and *Synechococcus* 7942 were grown in an MC-1000 multicultivator (Photon Systems Instruments) to an OD_730_ of 0.5. *Synechococcus* 2973 was grown at 900 μmol m^−2 ^s^−1^ light, and *Synechococcus* 7942 was grown at 400 μmol m^−2^^ ^s^−1^ light. Two 1-ml samples were sealed in 13-ml Hungate tubes with rubber septa and sparged with 3% CO_2_ for 5 min. One tube for each strain was placed on its side on a shaker under its respective white light-emitting diode (LED) illumination (900 or 400 μE m^−2 ^ s^−1^) at 38°C for 1 h after which time total CO_2_ was measured by gas chromatography (GC). The second tube was measured immediately without incubation to determine initial CO_2_. Total CO_2_ was calculated as the sum of the dissolved plus gaseous CO_2_ within a sealed system. Total CO_2_ of the system was determined after injecting 100 μl of 10 N HCl into the tube to force dissolved CO_2_ out of solution, followed by quantification of the headspace CO_2_ content on an HP 5980 gas chromatograph under the following conditions: temperature of 100°C; carrier gas, helium at a flow rate of 40 ml min^−1^; using a Porapak N column and thermal conductivity detector (TCD). CO_2_ uptake was determined as follows: initial total CO_2_ − final total CO_2_.

### Oxygen evolution.

Cells were harvested during log growth and adjusted based on equal cell numbers. The light-induced oxygen evolution was measured for 1 min at 38°C with a custom-built Clark-type electrode (18). For the light saturation curves, different neutral density filters were placed in front of the halogen light source to achieve different light intensities. For whole-chain O_2_ evolution, NaHCO_3_ was added to 10 mM. For the measurements of PSII-mediated O_2_ evolution, we added potassium ferricyanide (FeCN) to 1.2 mM and 2,6-dichloro-*p*-benzoquinone (DCBQ) to 0.6 mM.

### Room temperature fluorescence kinetics.

The kinetics of chlorophyll *a* (Chl *a*) fluorescence and the fluorescence parameters *F*_*v*_/*F*_*m*_ (maximum quantum yield) of photosystem II (PSII) were measured using a double-modulation fluorescence fluorometer, FL-200 (Photon Systems Instruments, Brno, Czech Republic) (19). The instrument contained red LEDs for both actinic (20-μs) and measuring (2.5-μs) flashes and was used in the time range of 100 μs to 100 s. Data from both strains were double normalized at *F*_0_ and *F*_*m*_, which were set at a relative fluorescence of 0 and 1, respectively.

### PSI content.

Cultures were grown in MC-1000 multicultivators at 38°C, 5% CO_2_, and either 900 or 400 μmol m^−2^ s^−1^ light for *Synechococcus* 2973 and *Synechococcus* 7942, respectively. Cells were harvested during log phase, cultures were adjusted to have equal cell numbers, and chlorophyll content was recorded. Then, 10 μM 3-(3′,4′-dichlorophenyl)-1,1-dimethylurea (DCMU) and 20 μM dibromothymoquinone (DBMIB) were added to block both linear and cyclic electron flow and changes in the absorbance at 705 nm of P700^+^ were recorded for 5 s under saturating light on a JTS-10 pump probe spectrophotometer. A molar extinction coefficient for P700^+^ of 70 mM^−1^ cm^−1^ was used to calculate the PSI content from the maximum absorbance.

### Cytochrome *f* and plastocyanin kinetics.

Cytochrome *f* and plastocyanin redox kinetics were recorded on a JTS-10 pump probe spectrophotometer (BioLogic Science Instruments, Grenoble, France). Cells were grown to mid-log phase in an MC-1000 multicultivator at 38°C, 5% CO_2_, and either 900 or 400 μmol m^−2^ s^−1^ light for *Synechococcus* 2973 and *Synechococcus* 7942, respectively. After harvest, the cell samples were adjusted to equal cell numbers and then dark adapted for 3 min before measurements were taken. Absorbance changes due to cytochrome *f* and plastocyanin oxidation and reduction were probed with a measuring beam consisting of short pulses provided by a white LED, and light was filtered through a 10-nm bandwidth interference filter centered at 546 nm, 554 nm, 563 nm, and 573 nm in separate experiments. Long-pass 3-mm-thick BG39 filters were placed in front of the detectors to block the light from an actinic LED emitting in the 720-nm region. Continuous far-red actinic illumination of 5 s was interrupted by short (200-µs) dark pulses, during which detecting pulses from the white LED were delivered. The data sets collected from experiments with the four interference filters were then deconvoluted to generate cytochrome *f* and plastocyanin absorption signals.

10.1128/mBio.02327-17.4 TABLE S1  Glycogen content and accumulation rate in *Synechococcus* 2973. Download TABLE S1, DOCX file, 0.02 MB. Copyright © 2018 Ungerer et al. 2018 Ungerer et al. This content is distributed under the terms of the Creative Commons Attribution 4.0 International license.
