# Supplementary figures and images for: Adjustments to Photosystem Stoichiometry and Electron Transfer Proteins Are Key to the Remarkably Fast Growth of the Cyanobacterium Synechococcus elongatus UTEX 2973
Source: mBio. 2018 Feb 6;9(1):e02327-17. doi: 10.1128/mBio.02327-17 (PMC5801466; doi:10.1128/mBio.02327-17)

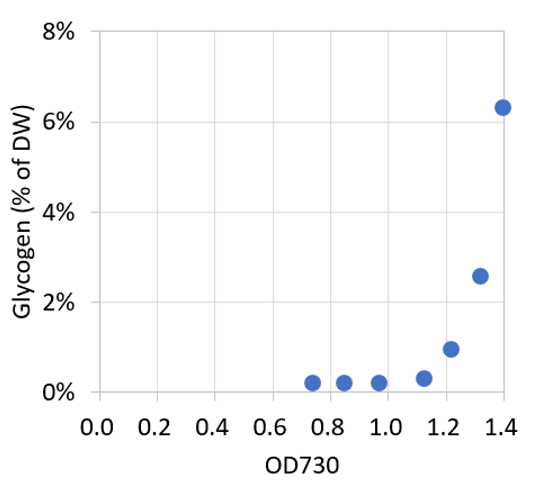

Supplement: FIG S1 [file mbo001183703sf1.tif]

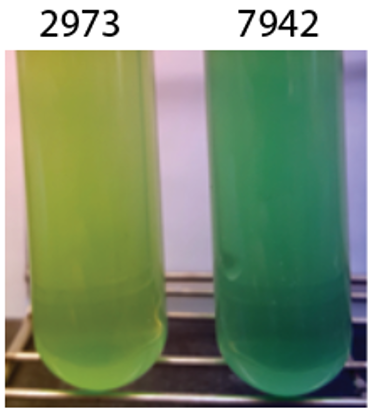

Supplement: FIG S2 [file mbo001183703sf2.tif]

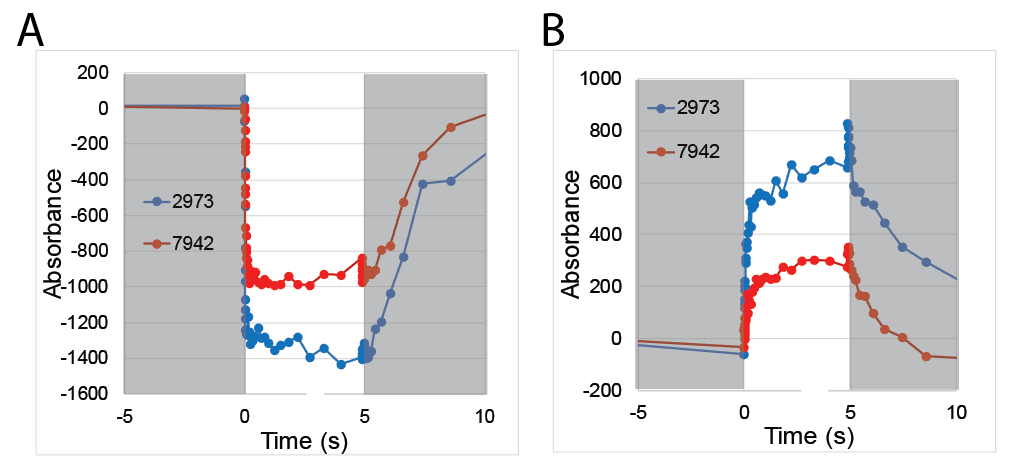

Supplement: FIG S3 [file mbo001183703sf3.tif]
