# Supplementary material for: Adjustments to Photosystem Stoichiometry and Electron Transfer Proteins Are Key to the Remarkably Fast Growth of the Cyanobacterium Synechococcus elongatus UTEX 2973
Source: mBio. 2018 Feb 6;9(1):e02327-17. doi: 10.1128/mBio.02327-17 (PMC5801466; doi:10.1128/mBio.02327-17)
Supplement: TABLE S1 [file mbo001183703st1.docx]

**Supplemental Figure 1.** Synechococcus 2973 glycogen content at different growth phase. DW, cell dry weight.

**Supplemental Figure 2.** Color difference between Synechococcus 2973 and Synechococcus 7942

**Supplemental Figure 3.** Absorbance changes for cytochrome f (A) and plastocyanin (B) with DCMU, DBMIB and MV. Shaded and unshaded areas represent times when the actinic light is off or on respectively

**Supplemental Table S1.** Glycogen content and accumulation rate in Synechococcus 2973.

| **Time (h)** | **Glycogen (mg L^-1^)** | **Accumulation rate (mg L^-1^ h^-1^)** |
| --- | --- | --- |
| 13 | 0.5 | - |
| 15 | 0.7 | 0.1 |
| 17 | 0.8 | 0.1 |
| 19 | 1.8 | 0.5 |
| 21 | 6.2 | 2.2 |
| 23 | 19.9 | 6.8 |
| 25 | 59.4 | 19.8 |
